# Organosilane for surface cleaning in intensive care units: protocol for a cluster randomized controlled trial with crossover

**DOI:** 10.62675/2965-2774.20250319

**Published:** 2025-06-30

**Authors:** Antonio Paulo Nassar, Claudia Vallone Silva, Camila Gosenheimer Righi, Isabella Lott Bezerra, Andrea de Carvalho, Ana Cristina Lagoeiro Patrocínio, Eduvirgens Maria Couto de Souza, Mirian Batista Rodrigues, Tiago Mendonça dos Santos, Luiz Felipe Valter de Oliveira, Ana Paula Christoff, Bianca Luise Teixeira, Bruno Adler Maccagnan Pinheiro Besen, Viviane Cordeiro Veiga, Alexandre Biasi Cavalcanti, Bruno Martins Tomazini, Adriano José Pereira

**Affiliations:** 1 Hospital Israelita Albert Einstein São Paulo SP Brazil Hospital Israelita Albert Einstein - São Paulo (SP), Brazil.; 2 BiomeHub Florianópolis SC Brazil BiomeHub - Florianópolis (SC), Brazil; 3 Universidade de São Paulo Faculdade de Medicina Hospital das Clínicas São Paulo SP Brazil Hospital das Clínicas, Faculdade de Medicina, Universidade de São Paulo - São Paulo (SP), Brazil.; 4 BP - A Beneficência Portuguesa de São Paulo São Paulo SP Brazil BP - A Beneficência Portuguesa de São Paulo - São Paulo (SP), Brazil.; 5 HCor-Hospital do Coração São Paulo SP Brazil HCor-Hospital do Coração - São Paulo (SP), Brazil.; 6 Hospital Sírio-Libanês São Paulo SP Brazil Hospital Sírio-Libanês - São Paulo (SP), Brazil.

**Keywords:** Organosilicon compounds, Disinfection, Desinfectants, Cross infection, Anti-bacterial agents, Antimicrobial stewardship, Critical care, Incidence, Intensive care units, Brazil

## Abstract

**Objective:**

To assess whether surface disinfection with organosilane in the intensive care unit reduces the occurrence of healthcare-associated infections.

**Methods:**

This multicenter, controlled, cluster-randomized trial includes 14 intensive care units in Brazil from November 2023 to December 2024. The local hygiene team of the included intensive care units will disinfect bed surfaces with organosilane or with usual care for 6 months, followed by a sequential crossover of another 6 months. The primary outcome is the incidence of healthcare-associated infections, specifically ventilator-associated pneumonia, central-line-associated bloodstream infections, and catheter-associated urinary tract infections. The secondary endpoints are the contamination of the environment by multidrug-resistant microorganisms (i.e., oxacillin-resistant *Staphylococcus aureus,* vancomycin-resistant *Enterococcus faecium,* carbapenem-resistant Enterobacter, *Pseudomonas,* and *Acinetobacter*), the incidence of specific infections (i.e., ventilator-associated pneumonia, central-line associated bloodstream infection, and catheter-associated urinary tract infection) and the cost of the patient's intensive care unit stay. We will enroll all adult patients admitted after the study begins in each participant's intensive care unit.

**Ethics and dissemination:**

The institutional review board of the coordinator center and each enrolled center approved the study protocol. We will disseminate the results in peer-reviewed journals and at scientific meetings, regardless of the study's outcome.

## INTRODUCTION

Healthcare-related infections (HAIs) are widespread events in intensive care units (ICUs). An international study to assess the prevalence of HAIs, involving 88 countries, showed that 12% of all patients admitted had an ICU-acquired infection, the presence of which was associated with higher mortality.^(1)^ A systematic review of 18 studies showed that admission to the ICU increases the odds of HAI acquisition by almost 4 times.^(2)^ In addition to increasing mortality, the acquisition of HAIs during an ICU stay may increase the length of hospital stay and, consequently, hospitalization costs.^(3)^

Contamination of hospital surfaces appears to play an important role in the transmission of HAIs. *Clostridium difficile*, methicillin-resistant *Staphylococcus aureus* (MRSA), vancomycin-resistant *Enterococcus,* and Gram-negative MR bacilli survive on surfaces for long periods.^(4,5)^ Several studies have shown that patients admitted to the ICU can acquire microorganisms from prior ICU bed occupants.^(6,7)^ Eradicating these pathogens is difficult with current environmental cleaning models. Even after adequate cleaning, environmental surfaces can quickly become contaminated again about 2 to 6 hours after the disinfection.^(8)^ Therefore, measures that can prolong the periods of suppression of microbial growth in the environment and thus reduce the occurrence of nosocomial infections may be a potential solution to this issue.

Organosilanes are a class of chemical substances composed of siloxane-based nanoparticles with antimicrobial properties. Organosilanes are stable in water and provide residual antimicrobial activity for surfaces that have already been cleaned using standardized procedures. Organosilanes do not affect microbial metabolism, which makes it difficult for resistance to develop. They produce a strong positive electrical charge, which breaks down the cell wall of microorganisms, acting against bacteria and fungi *in vitro.*^(9,10)^ To date, there have been few published studies evaluating the effects of organosilane.^(10)^ A randomized clinical trial carried out in a single ICU showed that organosilane-based cleaning reduced the number of colony-forming units by 65.9%. In comparison, traditional cleaning caused a reduction of only 35.1% over a 5-month study period.^(11)^ However, this study did not provide any data on the rate of patient infections. Therefore, the clinical impact of this intervention remains to be seen.^(12)^

This trial aims to assess whether surface disinfection with organosilane in the ICU reduces the occurrence of HAIs (i.e., ventilator-associated pneumonia [VAP], central-line-associated bloodstream infection [CLABSI], and catheter-associated urinary tract infection [CA-UTI]). Secondarily, it aims to assess whether surface disinfection with organosilane reduces surface colonization by multidrug-resistant organisms, whether it reduces specific HAIs (namely, VAP, catheter-associated primary bloodstream infection, and CA-UTI), and how the intervention impacts on ICU costs.

## METHODS

The study ORGanosilane for Surface CLEANing in Intensive Care Units (ORG-CLEAN) aims to assess whether surface disinfection with organosilane reduces the incidence of HAIs. The main characteristics of the ORG-CLEAN trial, according to World Health Organization (WHO) standards, are summarized in the synopsis table (Table 1).

**Table 1:** Synopsis (World Health Organization trial registration data set)

Data category	Information
Primary registry and trial identifying number	ClinicalTrials.gov ID NCT05673226
Date of registration in primary registry	January 5, 2023
Secondary identifying numbers	PROADI 25000.013526/2021-13
Development agency/funding source	Brazilian Ministry of Health (Institutional Development Programme of the Unified Health System)
Primary sponsor	Brazilian Ministry of Health
Secondary sponsor	*Hospital Israelita Albert Einstein*
Contact for public queries	Adriano José Pereira E-mail: Adriano.Pereira@einstein.br Phone: +55 (11) 2151-1500
Contact for scientific queries	Antonio Paulo Nassar Junior E-mail: antonio.nassar@einstein.br Phone: +55 (11) 2151-1500
Public title	Organosilane for surface cleaning in intensive care units
Scientific title	Organosilane for surface disinfection in intensive care units: a cluster randomized controlled trial with crossover
Countries of recruitment	Brazil
Health conditions/problems studied	Healthcare-acquired infections, intensive care unit
Interventions	Comparator: Surface disinfection with organosilane Control: Usual surface disinfection
Key inclusion and exclusion criteria	Inclusion: ICU clusters (all patients aged 18 or over admitted after the start of the study) Exclusion: ICUs that use any organosilane formulation for surface disinfection
Study type	Intervention/cluster Allocation: randomization Intervention design: crossover Masking: open Purpose: reduction in healthcare-acquired infections
Date of first enrollment	November 6, 2023
Sample size	14 clusters, approximately 13,000 patients
Recruiting status	Recruiting
Primary outcome	Incidence of healthcare-acquired infections
Secondary outcomes	− Occurrence of contamination of environmental surfaces by multidrug-resistant microorganisms; − Occurrence of the following HAIs: ventilator-associated pneumonia, catheter-associated urinary tract infection, and central line-associated bloodstream infections; − Evaluation of the economic impact of using organosilane.
Ethics review	First approved by *Hospital Israelita Albert Einstein* Institutional Review Board on 20 December 2021 (CAAE 52639521.1.1001.0071) and in each participating center after that (supplementary file). *Hospital Israelita Albert Einstein* Institutional Review Board. Phone: +55 (11) 2151-3729 E-mail: cep@einstein.br

ICU - intensive care unit; HAI - healthcare-related infections.

We used the Recommendations for Interventional Trials (SPIRIT) guidelines for this report.^(13)^ The final report will follow the Consolidated Standards of Reporting Trials (CONSORT) statement and its extension for cluster randomized trials^(14)^ and the Consolidated Health Economic Evaluation Reporting Standards 2022 (CHEERS 2022).^(15)^ The trial was registered with ClinicalTrials.gov (NCT05673226) before the inclusion of the first patient.

### Study setting

We propose a cluster study with a crossover in ICUs participating in the IMPACTO MR platform.^(16)^ The research team initially selected 16 ICUs, but two ICUs withdrew from the trial after randomization and before initial patient recruitment.

### Eligibility criteria

We include all patients aged 18 or over admitted to the ICUs participating in this clinical trial. All the participating ICUs are already part of the IMPACTO MR platform. We excluded ICUs that use any organosilane formulation for surface disinfection.

### Randomization

Specific computer software generates randomization codes sequentially, stratified by ICU size (up to ten beds *versus* more than ten beds), in a 1:1 ratio, assigning ICUs to either the intervention or the usual disinfection group. We randomized each unit to perform either usual surface disinfection or organosilane disinfection for 6 months.

### Blinding

The hygiene personnel applying the intervention cannot be blinded, as they will know which product they are using for disinfection. However, the statistician responsible for analyzing the data will be blinded to the study intervention.

### Intervention and usual care

The professionals from the hygiene and cleaning teams of the participating centers and their leaders received remote online training, which was replicated several times by the coordinating center with the support of a specialist nurse from the study.

During the 6 months of the study intervention, the hygiene/cleaning team at the participating centers proceeds with the terminal cleaning of the vacant bed using detergent and then applies the organosilane to it, aerosolizing the room from the back to the door and from top to bottom. After this process, the person responsible for the application moistens the cloth with organosilane and then rubs it on highly touched surfaces, such as furniture (e.g., the bed, chair, bedside table, gauze panel, communication panel, IV stand, dining table, and other furniture). The hygiene team uses organosilane both in single-bed and multi-bed ICUs whenever there is a need for terminal cleaning of the bed when the patient is discharged. During the intervention period, the hygiene team completed a form for each terminal cleaning performed using organosilane. This form assessed the length of time the patient spent in the ICU, whether they were under precautions or isolation, the time of day, and the total time of completion of the process.

During the 6 months of usual care, the hygiene/cleaning team disinfects the beds and environment according to their established protocols, using standard products.

**Figure 1:**
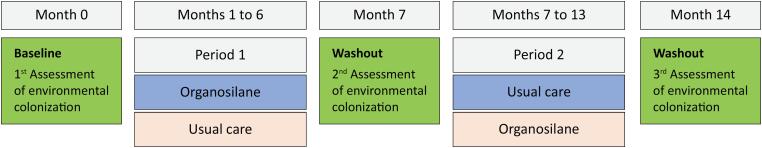
Trial timeline, interventions, and microbiological data collection.

After a 1-month washout period, the units previously randomized to organosilane crossover back to their usual disinfection regimen, while the units randomized to usual disinfection will receive the organosilane intervention (Figure 1).

### Outcomes

The primary outcome is the incidence of HAIs (i.e., VAP, CLABSI, and CA-UTI) during the 6-month study period, as diagnosed according to the criteria of the *Agência Nacional de Vigilância Sanitária* (ANVISA)^(17)^ (Table 2). The secondary endpoints are the contamination of the environment by multidrug-resistant microorganisms (i.e. oxacillin-resistant *Staphylococcus aureus,* vancomycin-resistant Enter*ococcus faecium,* carbapenem-resistant Enterobacter, *Pseudomonas* and *Acinetobacter*) at the end of each phase of the study; the incidence of each specific monitored HAI (VAP, CLABSI, and CA-UTI) and the cost of the patient's ICU stay.

**Table 2:** *Agência Nacional de Vigilância Sanitária* diagnostic criteria for healthcare-related infections

Healthcare-associated infection	ANVISA diagnostic criteria
Ventilator-associated pneumonia	Patient with two or more consecutive days of invasive mechanical ventilation use with no underlying heart or lung disease ***AND*** One of the following findings on thorax imaging tests, whether new, persistent, or progressive: − Infiltrate − Opacification/consolidation − Cavitation − Pneumatocele ***AND*** At least one of the signs and symptoms: − Fever (temperature: > 38ºC), with no other associated cause − Leucopenia (< 4,000 cells/mm3) or leucocytosis (> 12,000 cells/mm3) − Altered level of consciousness, with no other apparent cause, in patients ≥ 70 years ***AND*** At least two of the signs and symptoms: − Appearance of a purulent secretion or a change in the characteristics of the secretion or increased respiratory secretion or increased need for aspiration − Apnea, tachypnea, dyspnea, or cough (new or worsening episode) − Auscultation with wheezing, snoring, or rales (new or worsening episode) − Worsening of gas exchange, desaturation, increased oxygen demand, or increased ventilatory parameters for at least 2 days
Central line-associated bloodstream infections	− Patient using a central catheter for more than two consecutive days ***AND*** − Presents a pathogenic bacterial or fungal microorganism identified from one or more blood samples obtained in a hemoculture ***OR*** − Identified genus and species, or at least genus, by validated non-culture-based test methods ***AND*** − The identified microorganism is not related to another infectious focus
Catheter-associated urinary tract infection	− Patient using an indwelling bladder catheter for more than two consecutive days ***AND*** − Has at least one of the following signs and symptoms, with no other recognized causes: − Fever (T > 38ºC) − Suprapubic pain or discomfort − Lumbar pain or discomfort − Hematuria − Urinary urgency − Increased urinary frequency − Dysuria ***AND*** − Positive urine culture with a maximum of two bacterial species with a colony count ≥ 10^5^ CFU/mL

ANVISA - *Agência Nacional de Vigilância Sanitária*; T - temperature.

### Data collection

#### Clinical data

We will extract patient data from the Epimed system,^(18)^ which provides data in a manner that makes it impossible to identify patients. The data collected consists of sex and age, ICU admission data (date of admission to the ICU and hospital, comorbidities, severity measured by the Simplified Acute Physiology Score 3 (SAPS 3) and Sequential Organ Failure Assessment (SOFA) scores, type of ICU admission (clinical, elective surgical, or unplanned surgical), reason for admission, use of a central venous catheter, mechanical ventilation, and indwelling bladder catheter; development of HAIs, microbiological cultures, vital status and date of ICU and hospital discharge.

#### Microbiological data

To assess environmental contamination, trained professionals from the participating centers collect samples from 30% of the beds in each participating ICU from the following surfaces: bed rails, monitor and infusion pump buttons, and dining tables or procedure tables. The professionals collect the samples using specific flocked swabs, pre-moistened with a sterile saline solution.

The collection of samples takes place before the start of the study, at the end of the first study period (month 7), and at the end of the second study period (month 14) (Figure 1). The professionals responsible for collecting the samples send them in a stabilizing solution (BiomeHub, Brazil),^(19)^ Brazil for ambient temperature transport, to the sample processing laboratory (BiomeHub, Brazil) within 30 days of collection.

The study laboratory analyzes the environmental microbiome, which identifies the set of microorganisms in each location. This technique consists of amplifying the V3/V4 hypervariable region of the 16SrRNA gene and preparing libraries for second-generation sequencing using the Illumina platform.^(19)^ Brazil The Quantitative Insights into Microbial Ecology software (QIIME2 v. 2020.11) is used to analyze the sequencing data. Furthermore, the study laboratory will perform an analysis of antimicrobial resistance genes relevant to the Brazilian scenario using real-time polymerase chain reaction.^(20)^ The remaining sample volume will be stored at -20ºC during the study period.

#### Cost data

The research team will carry out a cost-effectiveness analysis of the intervention. Costs will be assessed from the hospital's perspective. The time horizon for costs and outcomes will be the patient's ICU stay. We will measure costs using a calculator under development, based on the cost data collected in the sub-study of costs within the IMPACTO MR platform.^(21)^ The collected data on costs consist of ICU staff, depreciation, electricity, water, telephone, internet, medical gases, overhead, office supplies, drugs, medical supplies, hemodialysis, blood transfusions, laboratory tests, and imaging tests. The researchers will adjust the costs by the Extended National Consumer Price Index (IPCA -*Índice Nacional de Preços ao Consumidor Amplo*) of the *Instituto Brasileiro de Geografia e Estatística* (IBGE) at the time of the cost analysis.^(22)^

#### Data collection and management

Trained healthcare workers collect data in each participating center using the Epimed system. The local infection control committee provides data on HAIs, and the data collectors enter it into a developed Research Electronic Data Capture system (REDCap, USA) via the internet, hosted on a server at *Hospital Israelita Albert Einstein* in São Paulo, Brazil. Access to all documents is controlled by user and password. To ensure data quality, we perform the following procedures: all professionals responsible for data collection are trained before the beginning of the trial; a researcher assistant from the coordinating center is responsible for solving any questions about data collection; a researcher from the coordinating center monitors the records of bed disinfection carried out by the hygiene team with the ICU discharges generated in the Epimed system weekly; the coordinating center assigns a researcher assistant to monitor the study participating centers; all the information obtained during the monitoring visits is confidential.

### Data monitoring

#### Interim analysis

Since the study's intervention is conducted in the environment and not directly on patients, we do not anticipate substantial risks to the study's performance. Therefore, we did not plan interim analyses. Adverse events are not expected to occur, but local researchers, data assistants, and local clinicians may report them. In any case, the research team assesses adherence to the intervention monthly by monitoring the exact number of bed disinfections after discharge, the volume of organosilane used (in mL), and the number of HAIs reported.

#### Auditing

Trial conduct is subjected to audit by Einstein Research Integrity Committee, at any time, independently of the institutional review board (IRB) and research team.

#### Sample size

We based the sample size calculation on a baseline incidence of HAIs of 9.88%, as indicated by preliminary data from a sample of the IMPACT MR platform cohort study conducted from 2018 to 2020.^(23)^ Assuming 556 patients per unit over 6 months, with a within-cluster within-period correlation of 0.01, a within-cluster between-period correlation of 0.008, a significance level of 5%, and a power of 80%,^(24)^ the study required a total of 14 participating units to detect a relative reduction of 20.2% (equivalent to an absolute reduction of 2%) in HAI rates due to the intervention. The estimated total number of patients across all participating units is 15,558.

After establishing the initial 14 centers, consideration was given to include two additional centers, bringing the total to 16. If we included these additional centers in the sample size calculation while maintaining all other parameters constant, it would require 411 patients per cluster per period, resulting in a total of 13,138 patients. However, the two additional centers ultimately did not participate in the study.

### Statistical analysis plan

We will present baseline data as the mean ± standard deviation (SD) for continuous variables with a normal distribution, or as the median [first quartile – third quartile] for non-normal distributions, and categorical variables as counts and percentages. We will report effect estimates for primary and secondary outcomes with 95% confidence intervals. All statistical tests will be two-sided, with a 5% significance level.

We will conduct all statistical analyses using R software version 4.2.2 or higher (R Core Team, 2022). We will not perform imputation for missing data on outcomes; all outcomes will be analyzed using complete case analysis.

The primary outcome analysis will estimate the risk ratio (RR) of HAIs between the intervention and control arms using a mixed logistic regression model. This model will incorporate random effects for clusters and cluster-period effects while adjusting for SAPS 3 and ICU size. If more than 5% of the SAPS 3 data are missing, we will apply multiple imputations under the assumption that the data are missing at random (MAR). We will proceed to the imputation using multivariate imputation by chained equations (MICE) based on patient characteristics (age, type of admission, and SOFA scores) using the ‘mice’ R package.^(25)^

As a sensitivity analysis for the primary outcome, we will use a complete-case analysis with the same mixed logistic regression model.

We will conduct a subgroup analysis of the primary outcome based on the type of admission, categorized as medical, elective surgical, and unplanned surgical.

We will analyze secondary outcomes using the same mixed logistic regression model used for the primary outcome.

### Ethics and dissemination

The *Hospital Israelita Albert Einstein* first approved the ORG-CLEAN trial on December 20, 2021 (CAAE 52639521.1.1001.0071), and subsequently, in each participating center, following Brazilian legislation. A specialist in the regulatory process oversees and supports the local process.

Any modifications in the protocol that might affect the development of the study and its potential benefits or safety, including changes in the objectives, design, or relevant management aspects, require amendments to the protocol, which must be submitted to the IRB of the coordinating center and all the IRBs at the participating centers for proper approval.

#### Consent form

Since this is an environmental intervention, rather than an individual-based one, we submitted a consent form for each of the initial 16 participating units, which was approved by the IRB of the coordinating center and the participating centers. There is no interference in the treatment of the patients included, which is entirely at the discretion of the ICU teams involved in the study.

#### Data confidentiality

Patient data is all anonymized, and each patient has a different entry number in the data system. Numbers also identify the centers. Locked systems store the data, and the identities of the participating patients are always kept anonymous.

The coordinating center will store all the study's information on magnetic media. Only the data manager and the statistician will access the patient-level data.

#### Harms

The intervention tested works by disinfecting the environment when the bed is vacant so that the patient does not encounter the sanitizer in use. There is no change in the treatment given to patients during the study.

#### Dissemination policy

The Steering Committee commits to publishing the study results in a manuscript published in an indexed medical journal.

#### Trial status

This manuscript presents the protocol for the ORG-CLEAN trial (original V.1.0, approved on December 20, 2021). The study commenced on November 6, 2023. At the time of manuscript submission, data collection for the trial was ongoing and was scheduled to be completed by the end of 2024.

### Declaration

The trial sponsor (*Hospital Israelita Albert Einstein*) and the trial funder (Brazilian Ministry of Health) had no role in the study design, data collection, management, analysis, and interpretation; the writing of the report; or the decision to submit the report for publication.
